# Perceptions of ultra processed food are associated with strategies for identifying healthy foods in online survey of adults living in Vermont

**DOI:** 10.3389/fpubh.2025.1679616

**Published:** 2025-11-04

**Authors:** Nick Rose, Jane Kolodinsky

**Affiliations:** ^1^Nutrition and Exercise Science, Bastyr University, Kenmore, WA, United States; ^2^Community Development and Applied Economics, University of Vermont, Burlington, VT, United States

**Keywords:** ultra processed food, food labeling, healthfulness perceptions, nutrition labeling, nutrition education, NOVA classification, clean eating, consumer nutrition

## Abstract

**Introduction:**

The public health effects of industrial food processing are being widely researched, in response to observational evidence linking ultra processed food (UPF) consumption with a range of poor health outcomes. While there has been global debate regarding whether UPF should be incorporated into dietary guidelines for the public (in addition to existing nutrient recommendations) there is a limited understanding of the degree to which consumers prioritize food processing attributes, relative to nutrient attributes (e.g., sugar, sodium, saturated fat) when making decisions about food and health. This study investigates how perceptions of UPFs relate to the criteria that consumers prioritize when evaluating food healthfulness, including both nutrient-based and non-nutrient-based attributes.

**Methods:**

An online survey was conducted in 2022 with a sample of 671 adults residing in a northeastern U.S. state. The survey assessed perceptions of UPFs, including efforts to reduce UPF consumption, and the importance assigned to various nutrient (sugar, fat, sodium, and food groups) and non-nutrient (kitchen ingredients, minimal processing, and production practices) criteria in determining food healthfulness. Demographic variables included age, gender, income, education, race/ethnicity, and presence of children in the household. Multivariate logistic regression analyzed associations between UPF perceptions and healthfulness criteria, reported as adjusted odds ratios (AORs).

**Results:**

A slight majority (52%) of respondents were familiar with the term “ultra-processed foods,” with awareness significantly higher among females (*p* < 0.05). Twenty-one percent of respondents selected all nutrient criteria as indicators of healthfulness, while 79.1% selected at least one non-nutrient criterion. Respondents actively trying to reduce UPF consumption (33.5%) were less likely (*p* < 0.05) to prioritize nutrient-based criteria and more likely to prioritize non-nutrient factors, including kitchen ingredients (AOR: 1.6, 95% CI: 1.1–2.4), processing (AOR: 3.0, 95% CI: 2.0–4.5), and production practices (AOR: 1.8, 95% CI: 1.2–2.6).

**Discussion:**

Findings suggest that perceptions of UPFs shape how consumers define healthy food, with many favoring non-nutrient criteria related to processing and production over conventional nutrient profiles. This shift in consumer perspective highlights the importance of incorporating processing-related information into public health communication and food labeling strategies.

## 1 Introduction

The relationships between diet and health have historically been viewed and described based on nutrient attributes (sugar, sodium, saturated fat, and calories); however, the focus of nutrition research has shifted over the past decade to also include assessments of food ingredients and the extent of processing (1–3). Much of this research cites the Nova classification system which differentiates foods into four categories based on the extent of processing and differentiates ultra-processed foods (Nova Group 4) from other processed foods (Groups 1–3) by the inclusion of cosmetic additives (flavors, sweeteners, preservatives, emulsifiers, etc.) and very little, if any, whole food (2, 4).

Ultra-processed foods are a controversial category of industrial formulations which are “unable to be made in home kitchens” (5) yet contribute the majority of calories consumed by children (6), adolescents (7), and adults (8) in the United States (U.S.). While there has been considerable debate about whether the concept of ultra-processing should be incorporated into national dietary guidelines in the U.S. (9–13) there is a limited understanding of the degree to which the public prioritizes nutritional attributes compared to non-nutritional attributes (including the extent of industrial processing) when making decisions about food and health. This study addresses these gaps to better understand U.S. consumer knowledge, attitudes, and behaviors related to the healthfulness of ultra-processed foods.

While the mechanisms linking ultra processed food (UPF) with poor health outcomes are poorly understood, numerous systematic reviews and meta analyses associate UPF consumption with a range of diet-related chronic diseases including heart disease, cancer, type 2 diabetes, metabolic syndrome, depression, gastrointestinal disorders, obesity, and all-cause mortality (14–18). An umbrella review of epidemiological meta-analyses found that UPF consumption was associated with 32 different health conditions leading the authors to conclude that efforts to target and reduce UPF exposure are needed to support human health outcomes (18). In response to this body of observational evidence, the topic of ultra-processed food has been incorporated into the dietary guidelines of several nations (13) including Brazil where the guidelines explicitly prioritize the consumption of “natural or minimally processed foods” and “freshly prepared meals” in addition to listing key nutrients of public health concern (19).

In the U.S., the Dietary Guidelines for Americans (DGA) have historically emphasized key nutrients of public health concern (and their associated food groups), however in development of the 2025 DGA the UPF concept was added (for the first time) to the list of topics for review by the DGA Scientific Advisory Committee (20). According to the U.S. Food and Drug Administration (FDA) website, the FDA is working along with the U.S. Departments of Agriculture (USDA) and Health and Human Services (HHS) “accelerating federal efforts to address the growing concerns around ultra-processed foods and the current epidemic of diet-related chronic disease that is plaguing America” (21). As more national dietary guidelines around the world incorporate advice about UPF, it is important to consider public understanding of the terms used and their capacity to act on advice provided (13). A 2022 survey of a nationally representative sample of U.S. adults reported that while only 24% of respondents had previously heard of the term “ultra processed” food, the health concerns linked to processed foods were a notable concern for many survey respondents (22). Respondents reported that the list of ingredients was the most common reference point for assessing the extent of food processing (22).

When making decisions about food and health the lay public demonstrates wide frameworks that incorporate assessments of a range of nutrient and non-nutrient factors (23–27). In addition to nutrient content, an individual’s healthfulness perception can be influenced by a range of factors including perceptions of the list of ingredients, organic status, product category and packaging characteristics, each of which can become incorporated into an individual’s health motivations (24). Lusk (25) reported that when defining “healthy” foods, approximately half of U.S. food consumers rely entirely on nutrient criteria, while the other half evaluate healthiness based on a broader range of factors, including food production, processing, and ingredient characteristics. When describing the criteria for use in defining “healthy” foods in a national dataset of public opinions on “healthy” food, Belarmino et al. (27), reported that nearly half of people cited attributes related to food processing and/or whole foods, while one third cited environmental sustainability dimensions, most often including considerations for agrichemicals and genetically engineered ingredients.

While survey research indicates the majority of food consumers report use of nutrition labels when food shopping, experimental methods have often reported lower rates of actual usage, as nutrition panels are not the only source of information relevant to consumer health. When tasked with evaluating the healthfulness of food packages in an online (experimental) environment, only 30% of respondents viewed the nutrition panels prior to making their decisions, indicating that healthiness perceptions were often made without even considering the food’s nutrient content (24). The impact of food processing on perceived healthiness was experimentally tested in an online survey of Swiss consumers who were presented with descriptions of foods that differed by processing level (28). Researchers found that the (perceived) degree of food processing was inversely correlated with perception of food healthiness, with strong effect sizes observed, leading the authors to conclude that “consumers have rather negative associations with food processing” (28). The authors also noted that consumer perceptions of processed foods were “very similar” to the Nova classification system for identifying UPF (28). Qualitative researchers have also commented on the “striking similarity” between Nova and the criteria individuals use for classifying food products in Brazil (29).

A cross-sectional study in Minnesota reported that when asked to define the characteristics of “healthy” foods, low-income consumers most often reported specific food groups (fruits, vegetables, grains, and meats), and a notable number of respondents defined healthy food based on being “natural” and or “fresh” (26). Freshness, naturalness, and minimal processing were identified as the most desirable attributes when considering food healthfulness in a global survey on healthy eating trends conducted by Nielsen Company (30). According to a 2017 systematic review on the importance of food naturalness to the public, “food naturalness is crucial” for the majority of consumers around the world, and “there is no reason to believe that the importance of naturalness will diminish in the future” (31). The recent trend of so-called “clean eating” has been described as “an approach to healthy eating which promotes the exclusion of processed foods” and an eating pattern that emphasizes “whole foods” (32). Clean eating was the most frequently cited popular diet by adults reporting practicing a specific diet in the US in 2019 and 2022 (33, 34) according to nationally representative surveys of US consumers.

Most U.S. consumers report use of food labels when evaluating food products in the marketplace, and more frequent use of nutrition labels is linked with healthier diets (35, 36). While the use of nutrition panels has been linked with healthier dietary outcomes, fewer studies have assessed consumer use of other label claims, and therefore it is unknown the degree to which prioritization of other types of food labels (including production and processing claims) might be linked with health or dietary outcomes (36). According to Monteiro (9), in the absence of labels, a practical way to identify UPF is to scan the list of ingredients on a food product and search for “food substances never or rarely used in home kitchens” such as high fructose corn syrup, or other food additives. The present study seeks to identify the presence of similar consumer strategies for identifying healthy foods in the U.S. marketplace (in the absence of dietary guidelines to reduce UPF intake), a concept that has not been previously tested and is relevant to current conversations on how best to educate the public about food and health.

The objectives of this cross-sectional analysis are to describe consumer perceptions of UPF and model the likelihood of prioritizing nutrient and non-nutrient attributes when evaluating food healthfulness, as predicted by self-reported perceptions of UPF. The specific aims of this study are: (i) to describe U.S. food consumers’ perceptions of UPF products, (ii) to describe the nutrient and non-nutrient criteria prioritized by consumers when evaluating the healthfulness of food in an online survey environment, and (iii) to analyze the associations between perceptions of UPF and the criteria prioritized when evaluating food healthfulness.

We hypothesize that those reporting attempts to reduce their UPF consumption will be less likely to prioritize the nutrient criteria, which are based on key messages in the 2020 DGA (sugar, sodium, fat, and food groups) and will instead be more likely to prioritize non-nutrient criteria when describing food-health relationships. The non-nutrient criteria tested are attributes related to the popular trend of so-called “clean” eating, which was the most popular diet in the U.S. during the year of the survey (2022). We also examine associations with demographic variables as previous research finds demographic variations in use of nutrition panels (35, 37) and other food labels when food consumers are making decisions related to their health. The Supplementary Figure S1 presents a conceptual overview of the study including the nutrient and non-nutrient indicators examined in the present study along with the general study hypothesis.

## 2 Materials and methods

### 2.1 Setting and participants

This cross-sectional on-line survey was conducted by the Center for Rural Studies at the University of Vermont in spring of 2022. A random sample was drawn from a commercially available email list. Data were collected using email-based outreach in March and April of 2022. Respondents identifying as current Vermont residents over the age of eighteen were eligible to participate. A total of 782 started the survey, and we present the results from the 671 respondents who fully completed the survey questions related to the variables of interest. We also present a comparison of the sample with census data, based on the US Census Bureau’s American Community Survey data from 2016 to 2020 for the state of Vermont. The study protocol was approved by the University of Vermont Institutional Review Board.

### 2.2 Survey instrument and variables

The questionnaire covered a variety of subject areas including a range of food system topics including personal use of food labels and attitudes toward innovative agricultural innovations including the reintroduction of hemp and genetically modified foods [results of which have been reported elsewhere (38, 39)]. Here we report respondents’ self-reported perceptions of ultra-processed foods, and the nutrient and non-nutrient criteria prioritized for making healthier food choices in the marketplace. Demographic profiling questions included gender, age, race/ethnicity, income, educational attainment, and having children in the home, and these were collected at the end of the questionnaire. We analyze and report unweighted data.

Following a series of questions regarding the use of food labels in the marketplace, and before any questions about perceptions of UPF, respondents were asked to identify the health-related criteria that they prioritized when selecting foods in the marketplace. Respondents were presented with a list of seven different criteria for evaluating the healthfulness of foods and were asked to select their three most important three criteria, reflecting the three most important attributes prioritized when evaluating the healthfulness of food products. The seven criteria included four nutrient-oriented attributes based on the key messages of the Dietary Guidelines for Americans (40) and three non-nutrient attributes which were developed based on the popular trend of “clean” eating (41, 42), the most frequently cited popular diet in a national consumer survey (22) in 2022, (the same year as the present study). The seven criteria were: *(i) quantity of added sugar, (ii) quantity of sodium, (iii) quantity, and or specific type of fat, (iv) food groups (grains, vegetable, fruit, meat, nuts), (v) ingredients list is similar to my kitchen, (vi) unprocessed, or minimally processed ingredients, (vii) production practices (use of pesticides, gmos).*

To assess perceptions of UPF, participants were asked to identify which statement best described themselves: *“(i) I have never heard of the term ultra-processed food; (ii) I have heard of ultra-processed food, and I am not concerned with the level of food processing of the foods that I but for my household; (iii) I have heard of ultra-processed food and I am concerned about the level of food processing in the foods I eat, but I have not made changes in the foods I buy for my household; (iv) I have heard of ultra- processed food and I am concerned about the level of food processing in the foods I eat and I have made changes in the foods I buy for my household; (v) do not know/unsure.”*

Respondents selecting “do not know/unsure” (*n* = 53) were combined with “I have never heard of UPF” as it was assumed that if someone did not know whether they had heard of UPF, then it was more likely that they had not heard of UPF, and it was unlikely that they were concerned about UPF and/or making changes to avoid UPF. Sensitivity analyses were performed to compare associations between variables in a second model (provided as Supplementary Table S1), which classified UPF perceptions as five categories (instead of four) by separately analyzing those who reported “do not know” and “never heard of” ultra processed food into separate response categories to examine potential differences between those reporting “unsure” and “never heard of” UPF groups and strengthen confidence in modeling.

### 2.3 Statistical analysis

Descriptive statistics are used to present the characteristics of the sample including perceptions of ultra-processed food (UPF), and the frequency of each criterion (nutrient and non-nutrient) prioritized when evaluating healthy foods. Chi-square analysis (Pearson) examines relationships between study variables including UPF perceptions and demographic characteristics (age, children in the home, educational attainment, annual income, gender, and race/ethnicity), as well as relationships between each healthfulness perception indicator (sugar, sodium, fat, food groups, ingredients, processing, and farming) and the same profiling characteristics. When evaluating the associations between age, income, and educational attainment and each healthfulness perception indicator, we report the results of Mantel–Haenszel linear trends. We report significant associations when *p* < 0.05, and also report significance levels (*p* < 0.05, *p* < 0.01, *p* < 0.001) when reporting significant associations.

To analyze the extent to which perceptions of UPF are associated with the criteria prioritized for evaluating the healthfulness of foods, we first performed a bivariate chi-square (Pearson) analysis that compared the frequency of respondents selecting each healthfulness perception indicator (sugar, sodium, fat, food groups, ingredients, processing, and farming), when grouped by perceptions of UPF (four distinct response categories). We report Cramer’s V as a measure of effect size for the associations between UPF perceptions and each healthfulness perception indicator.

To further examine the relationships between these variables, multivariate logistic regression analysis was used to estimate the likelihood of selecting each healthfulness perception indicator (sugar, sodium, fat, food groups, ingredients, processing, and farming), as predicted by perceptions of ultra-processed food (four categories), controlling for all demographic variables reported in this analysis. Multivariate logistic regression analyses are reported as adjusted (multivariate) odds ratios with 95% CIs, controlling for the potentially confounding effects of study variables. To test multicollinearity, we checked the variance inflation factor and tolerance to confirm acceptable values and to support the most accurate models. In modeling the relationships between perceptions of UPF and each healthfulness perception indicator, the reference category is set as those who reported never previously hearing of the term ultra-processed food (or unsure) to test the study hypothesis that those reporting efforts to avoid UPF are more likely to select non-nutrient attributes when evaluating food healthfulness than those who have never heard of UPF.

## 3 Results

A description of the sample is presented in Table 1 along with a comparison of how the sample compares with census data. The majority (87%) of respondents were 55 or older and reported attaining a college degree or higher (77%). Half (52%) of respondents reported earning $75,000/year annually or higher, and 16% reported having children in the home under the age of 18. Over half (57%) of respondents identified as female, 42% identified as male, and 1.9% reported non-binary/other genders.

**Table 1:** Characteristics of a sample of adults living in Vermont who completed an online survey in 2022, including comparisons with census data for the state of Vermont.

Variables	Sample	Census
	(*N* = 671)	ACS (Vermont)
	% (*n*)	% of adults
Gender		
Male	41.5 (269)	45.8%
Female	56.6 (367)	51.1%
Other	1.9 (12)	Unknown
Age		
18–34	2.7 (18)	27 7%
35–54	20.7 (139)	29.7%
55–74	63.2 (424)	33.2%
75+	13.4 (90)	9.3%
Kids in the home		
Yes	15.9 (105)	24.7%
No	84.1 (556)	75.3%
Educational attainment		
Up to high school	7.3 (48)	35.7%
Some college	16.3 (108)	20.5%
College graduate	76.5 (508)	43.8%
Annual income		
Less than $25,000/year	6.6 (39)	18.0%
$25,000–$49,999/year	18.3 (108)	21.2%
$50,000–$74,999/year	16.0 (94)	18.3%
$75,000–$99,999/year	21.2 (125)	13.9%
$100,000/year (or more)	37.9 (223)	28.5%
Race and ethnicity		
White	89.0 (597)	92.1%
Nonwhite	11.0 (74)	7.8%

The census data is based on the US Census Bureau’s American Community Survey (ACS) data from 2016–2020 for the state of Vermont.

### 3.1 Perceptions of ultra processed foods

Self-reported perceptions of ultra processed foods (UPF) are presented in Table 2. About half (48.0%) of online survey respondents reported that they had never heard of or were unsure as to whether they have heard the term “ultra-processed food.” Of the 52.0% of respondents who reported that they had previously heard of the term “ultra-processed food”—the majority (91%) reported that they were “concerned” about the levels of UPF in their diets. One in three respondents (33.5%) reported that they had made changes in the foods they purchase and consume, in attempting to reduce their consumption of UPF.

**Table 2:** Perceptions of ultra processed food, as reported in an online survey of adults (*N* = 671) living in Vermont in March/April 2022.

Perceptions of ultra processed food	% of respondents
“I have never heard of the term ultra-processed food” (*n* = 270)	40.2%
“I do not know/unsure whether heard of ultra processed food” (*n* = 52)^a^	7.8%
“I have heard of ultra processed food, and I am not concerned with the level of food processing of the foods that I buy for my household” (*n* = 32)	4.8%
“I have heard of ultra processed food, and I am concerned about the level of food processing in the foods I eat, but I have not made changes in the foods I buy for my household” (*n* = 92)	13.7%
“I have heard of ultra processed food, and I am concerned about the level of food processing in the foods I eat, and I have made changes in the foods I buy for my household” (*n* = 225)	33.5%

a This group was combined with respondents who reported that they had “never heard of the term ultra-processed” in statistical analysis.

Perceptions of UPF were not significantly different across any of the study variables in the bivariate analysis, with the exception of gender (Table 3). Females more often (*p* < 0.05) reported concern with food processing, and/or changes to reduce UPF consumption, while males more often reported that they had not heard of (or had heard of but were not concerned with) UPF.

**Table 3:** Descriptive characteristics of a sample of adults (*N* = 671) living in Vermont in 2022, grouped by perception of ultra-processed foods (UPF), as reported in an online survey.

Variables	Overall	Perceptions of ultra processed food (UPF)^a^				
	(*N* = 671)	Never heard of UPF	Not concerned about UPF	Concerned about UPF	Made changes to reduce UPF	sig.
	% (*n*)	48.0% (322)	4.8% (32)	13.7% (92)	33.5% (225)	
Gender						
Male/other	43.4 (281)	47.8 (149)	54.8 (17)	39.8 (35)	36.9 (80)	*p* = 0.040*
Female	56.6 (367)	52.2 (163)	45.2 (14)	60.2 (53)	63.1 (137)	
Age						
18–34	2.7 (18)	1.9 (6)	6.3 (2)	4.3 (4)	2.7 (6)	*p* = 0.143
35–54	20.7 (139)	18 (58)	18.8 (6)	28.3 (26)	21.8 (49)	
55–74	63.2 (424)	64.3 (207)	65.6 (21)	52.2 (48)	65.8 (148)	
75+	13.4 (90)	15.8 (51)	9.4 (3)	15.2 (14)	9.8 (22)	
Kids in the home						
Yes	15.9 (105)	14.3 (45)	25 (8)	20.7 (19)	14.9 (33)	*p* = 0.228
No	84.1 (556)	85.7 (270)	75 (24)	79.3 (73)	85.1 (189)	
Educational attainment						
Up to high school	7.3 (48)	9.1 (29)	6.5 (2)	7.6 (7)	4.5 (10)	*p* = 0.263
Some college	16.3 (108)	17 (54)	16.1 (5)	20.7 (19)	13.5 (30)	
College graduate	76.5 (508)	73.9 (235)	77.4 (24)	71.7 (66)	82.1 (183)	
Annual income						
Less than $25 k	6.6 (39)	5.8 (16)	3.4 (1)	5 (4)	8.8 (18)	*p* = 0.639
$25–50 k	18.3 (108)	21 (58)	6.9 (2)	16.3 (13)	17.2 (35)	
$50–75 k	16.0 (94)	17.4 (48)	17.2 (5)	18.8 (15)	12.7 (26)	
$75–100 k	21.2 (125)	19.9 (55)	27.6 (8)	21.3 (17)	22.1 (45)	
$100 k+	37.9 (223)	35.9 (99)	44.8 (13)	38.8 (31)	37.9 (80)	
Race and ethnicity						
White	89.0 (597)	87.9 (283)	84.4 (27)	93.5 (86)	89.3 (201)	*p* = 0.390
Nonwhite	11.0 (74)	12.1 (39)	15.6 (5)	6.5 (6)	10.7 (24)	

Reported *p* values represent differences in observed frequencies (across rows) between response categories (four categories), based on Pearson chi-square test (two sided). *Indicates significant associations (*p* < 0.05).

a The full descriptions of each UPF response category are listed in Table 2.

### 3.2 Strategies for identifying healthy foods

When selecting the criteria most prioritized for evaluating the healthfulness of foods, respondents most often reported: levels of added sugar (52.6% of respondents), followed by familiar ingredients (44.4%), minimal processing (42.8%), production/farming practices (42%), sodium content (41.3%), fat profile (37.3%), and food groups (36.0%). Less than one in four (20.9%) respondents selected all nutrient-oriented criteria (sugar, sodium, fat, and food groups). Most respondents selected at least one (36.7%) or two (33.3%) of the non-nutrient indicators (familiar ingredients, minimal processing, and farming/production practices), and 8.5% selected all non-nutrient indicators. The full description of each criteria is presented in Table 4, along with the frequency in the dataset, grouped by perception of UPF.

**Table 4:** Frequency of respondents selecting each nutrient and non-nutrient criteria for evaluating food healthfulness, grouped by perception of ultra-processed foods (UPF), as reported in an online survey of adults living in a small, northeastern U.S. state.

Criteria selected for describing healthy foods	Overall sample	Perception of ultra-processed food (UPF) categories^a^				
	(*N* = 671)	Never heard of UPF	Not concerned about UPF	Concerned about UPF	Made changes to reduce UPF	sig. *χ^2^*(3)
	% (*n*)	48.0% (322)	4.8% (32)	13.7% (92)	33.5% (225)	*Cramer V*
Nutrient criteria						
Quantity of added sugar	52.6 (353)	57.1 (184)	50.0 (16)	58.7 (54)	44.0 (99)	0.1269*
Quantity of sodium	41.3 (277)	48.1 (155)	40.6 (13)	43.5 (40)	30.7 (69)	0.1587***
Quantity, and or specific type of fat	37.3 (250)	42.9 (138)	18.8 (6)	47.8 (44)	27.6 (62)	0.1830***
Food groups (grains, vegetable, fruit, meat, nuts)	35.9 (241)	37.6 (121)	50.0 (16)	38.0 (35)	30.7 (69)	0.0947
Non-nutrient criteria						
Ingredients list is similar to my kitchen	44.4 (298)	39.8 (128)	50.0 (16)	33.7 (31)	54.7 (123)	0.1596***
Unprocessed, or minimally processed ingredients	42.8 (287)	32.9 (106)	46.9 (15)	37.0 (34)	58.7 (132)	0.2364***
Production practices (use of pesticides, gmos)	41.7 (280)	37.3 (120)	40.6 (13)	32.6 (30)	52.0 (117)	0.1523***

The percentages for the criteria for describing healthy foods add up to over 100% because respondents selected three of seven provided criteria, when asked “when evaluating the healthfulness of foods in the marketplace, which of the following criteria do you prioritize?” followed by a list of these seven criteria. Cramer’s V is an estimate of effect size, with values of 0.06 interpreted as a small effect, and values of 0.17 interpreted as a medium/moderate effect, and values greater than 0.29 interpreted as a large effect size. Reported *p* values represent differences in observed frequencies (across rows) between response categories (four categories) for each healthfulness perception criteria, based on Pearson chi-square test (two-sided). *p < 0.05, **p < 0.01, ***p < 0.001.

a The full descriptions of each UPF response category are listed in Table 2.

### 3.3 Associations between healthfulness perception indicators and study variables

The healthfulness perception indicators were associated with several study variables in bivariate (chi-square) analysis. Females more often (*p* < 0.05) prioritized all three of the non-nutrient criteria, including kitchen ingredients (50% vs. 40%), minimal processing (48% vs. 40%) and farming practices (49% vs. 35%) when evaluating the healthfulness of food products, when compared to male and non-binary respondents (who were grouped together for analysis). Those with children in the home less often (*p* < 0.05) prioritized the nutrient criteria sodium (25% vs. 45%) and fat (25% vs. 40%), and more often (*p* < 0.05) prioritized food processing (51% vs. 41%). Age categories were also associated with criteria selection, with older respondents appearing to more often (*p* < 0.001) prioritize sodium and fat, and less often prioritize ingredients (*p* < 0.001) and processing (*p* < 0.01). Higher levels of educational attainment were associated with higher frequency of prioritizing food processing (*p* < 0.01) while income and race/ethnicity were not associated with any healthfulness perception indicators. Added sugar was the most frequently selected criteria, and was the only criteria that did not differ significantly when grouped by any demographic variables.

### 3.4 Associations between healthfulness perception indicators and perceptions of ultra processed food

Perceptions of ultra-processed foods (four response categories) were significantly associated with six of the seven healthfulness perception indicators in bivariate (chi-square) analysis (presented in Table 4). As hypothesized, those who reported making changes to reduce purchase and consumption of ultra-processed foods (33.5% of respondents) more often (*p* < 0.001) selected kitchen ingredients (54.7% vs. 44.1% overall), minimal processing (58.7% vs. 42.4% overall), and farming practices (52% vs. 42% overall) than other respondents. As expected, these respondents less often selected the nutrient criteria added sugar (*p* < 0.05), sodium (*p* < 0.001), and fat (*p* < 0.001). Medium effect sizes were observed for most of the healthfulness perception criteria including minimal processing (Cramer’s *V* = 0.2364) and familiar ingredients (*V* = 0.1596). “Food groups” was the only criteria that was equally prioritized across all UPF perception categories. These relationships were further tested in multivariate analysis, adjusting for the effects of demographic study variables.

In multivariate logistic regression analyses, perceptions of ultra-processed foods remained significantly associated with each healthfulness perception indicator after controlling for all study variables with small (0.02–0.049) to medium (0.05–0.09) effect sizes noted. When compared to those who had never heard of UPF (48% of sample), those who reported efforts to reduce their purchase/consumption of UPF (34% of sample) were three times more likely to prioritize minimal processing (AOR: 3.0, 95% CI: 2.0–4.5), 76% more likely to prioritize farming/production practices (AOR: 1.76, 95% CI: 1.2–2.6), and 60% more likely to prioritize familiar ingredients (AOR: 1.6, 95% CI: 1.1–2.4) after controlling for all study variables. Table 5 presents the full results of multivariate logistic regression modeling of the relationships between the odds of selecting each criterion for describing food health relationships, as predicted by perceptions of ultra-processed food, and additional study variables.

**Table 5:** Summary results of multivariate logistic regression analyses, modeling the odds of selecting each healthfulness perception indicator, as predicted by perceptions of ultra-processed foods and study variables (*N* = 671).

Adjusted odds ratios (and 95% confidence intervals) for selecting each healthfulness perception indicator^a^							
Variables	Nutrient-criteria				Non-nutrient criteria		
	Sugar	Sodium	Fat	Food groups	Ingredients	Processing	Production
Ultra-processed food (UPF) perceptions							
Never heard of UPF (ref)	1.0 (ref)	1.0 (ref)	1.0 (ref)	1.0 (ref)	1.0 (ref)	1.0 (ref)	1.0 (ref)
Not concerned about UPF	0.92 (0.42–2.0)	1.12 (0.49–2.5)	0.24 (0.09–0.67)*	1.36 (0.60–2.9)	1.37 (0.62–3.0)	1.70 (0.76–3.8)	1.02 (0.46–2.3)
Concerned about UPF	1.00 (0.60–1.7)	0.99 (0.58–1.7)	1.35 (0.79–2.3)	1.04 (0.58–1.7)	0.67 (0.39–1.2)	1.12 (0.65–1.9)	0.73 (0.42–1.3)
Made changes to reduce UPF	0.66 (0.45–0.97)*	0.50 (0.33–0.75)**	0.49 (0.32–0.74)*	0.65 (0.43–0.97)*	1.61 (1.1–2.4)*	3.00 (2.0–4.5)***	1.76 (1.2–2.6)**
Control variables (and reference categories)							
Female (ref. male/other)	0.96 (0.68–1.4)	0.66 (0.45–0.89)	0.61 (0.42–0.89)	1.07 (0.73–1.5)	1.45 (0.99–2.1)	1.15 (0.75–1.6)	1.48 (1.0–2.4)**
Age 18–34 (ref. 55–74)	1.10 (0.40–3.0)	0.33 (0.09–1.2)	0.47 (0.13–1.5)	2.05 (0.75–5.6)	1.22 (0.43–3.4)	1.80 (0.61–5.3)	1.12 (0.41–3.2)
Age 35–54 (ref. 55–74)	0.86 (0.53–1.4)	0.63 (0.36–1.1)	0.52 (0.30–0.90)*	1.46 (0.90–2.4)	1.80 (1.1–2.9)*	0.87 (0.53–1.5)	1.47 (0.91–2.4)
Age 75+ (ref. 55–74)	1.06 (0.63–1.8)	2.35 (1.4–4.0)**	0.95 (0.57–1.6)	0.87 (0.50–1.5)	0.64 (0.37–1.1)	0.60 (0.34–1.0)	1.06 (0.63–1.8)
Education: high school (ref. college graduate)	1.25 (0.61–2.6)	2.87 (1.3–6.1)**	1.03 (0.48–2.2)	0.83 (0.39–1.8)	0.52 (0.23–1.1)	0.57 (0.23–1.1)	0.97 (0.46–1.4)
Education: Some college (ref. college graduate)	1.17 (0.73–1.9)	1.78 (1.0–2.9)*	1.19 (0.71–1.9)	0.77 (0.46–1.3)	1.38 (0.85–2.3)	0.46 (0.27–0.78)**	0.78 (0.48–1.3)
Children in home (ref. no children)	1.22 (0.72–2.1)	0.61 (0.33–1.1)	0.79 (0.44–1.4)	0.99 (0.58–1.7)	1.07 (0.61–1.8)	1.54 (0.89–2.6)	0.85 (0.48–1.4)
Variance explained, R-squared (%)							
UPF only	0.012*	0.019*	0.026*	0.006	0.019*	0.041***	0.017*
UPF plus Covariates	0.011	0.092***	0.065***	0.027	0.053***	0.075***	0.035*

Data are reported as Adjusted Odds Ratios (and 95% CI), controlling for all study variables, and reference categories for each comparison are listed in parentheses. Reported *R*-squared is Nagelkerke (Pseudo *R*-squared). *Indicates significant associations (*p < 0.05*) in logistic regression, after controlling for all demographic variables reported in table, as well as additional study variables, including race/ethnicity and annual income. *p < 0.05, **p < 0.01, ***p < 0.001.

a Full description of the healthfulness perception criteria, as presented to each respondent: *“quantity of added sugar”; “quantity of sodium”; “quantity, and or specific type of fat”; “food groups (grains, vegetable, fruit, meat, nuts)”; “ingredients list is similar to my kitchen”; “unprocessed, or minimally processed ingredients”; “production practices (use of pesticides, gmos)*”.

Some of the demographic variables remained associated with the healthfulness perception criteria, including age, gender, and educational attainment. The oldest respondents (75+) were twice as likely to prioritize sodium (AOR: 2.35, 95% CI: 1.4–4.0) when compared to largest group of respondents (55–74 y/o), and females were less likely to prioritize fat (AOR: 0.61, 95% CI: 0.4–0.9) or sodium (AOR: 0.66, 95% CI: 0.5–0.9) and more likely to prioritize farming practices (AOR: 1.48, 95% CI: 1.0–2.4), but not processing or ingredients. When compared to the highest education category (largest group), those with some college education were half as likely to select processing (AOR: 0.46, 95% CI: 0.3–0.8), and 78% more likely to select sodium (AOR: 1.78, 95% CI: 1.0–2.9).

## 4 Discussion

We report that half (52%) of respondents had previously heard of the term “ultra-processed food” and the majority (91%) of those who had heard of the term were “concerned” with the level of processing in the foods they eat. Familiarity with the term “ultra-processed food” was higher in our study than the 25% reported in a national survey conducted by the International Food Information Council (IFIC) the same year (22).

One third (33.5%) of respondents explicitly reported efforts to reduce their purchase and consumption of UPF, and these respondents were less likely (*p* < 0.05) to prioritize sugar (AOR: 0.66, 95% CI: 0.45–0.97), sodium (AOR: 0.50, 95% CI: 0.33–0.75), and fat (AOR: 0.49, 95% CI: 0.32–0.74), and instead prioritized minimal processing (AOR: 3.01, 95% CI: 2.0–4.5), transparent production practices (AOR: 1.76, 95% CI: 1.2–2.6), and familiar ingredients (AOR: 1.61, 95% CI: 1.1–2.4) when describing the relationships between food and health. These findings support the study hypotheses (Table 6) and demonstrate the presence of consumer strategies for identifying and avoiding UPF in the marketplace.

**Table 6:** Associations between self-reported efforts to reduce ultra-processed food (UPF) purchase and consumption and the criteria prioritized when describing food and health relationships, in a sample of adults (*N* = 671) summarized for each hypothesis.

Hypotheses and hypotheses directions	Independent variable, and comparison category	Dependent variables	Results of multivariate logistic regression, controlling for study variables, reported as Odds Ratios (95% CI), adjusted odd ratios (AORs)^a^	Summary of results
H1: adults reporting efforts to reduce UPF are more likely to prioritize non-nutrient criteria when evaluating food healthfulness.				
H1a: more likely to prioritize *“Ingredients list similar to my home kitchen”*	*“Made changes in the foods I buy”* to reduce ultra-processed food, compared to *“never heard of”* ultra processed food	Frequency of prioritizing:	OR: 1.83 (1.30–2.58)** AOR: 1.61 (1.09–2.38)*	*H1* supported: adults reporting both concern about food processing and efforts to reduce UPF purchase (33.5% of sample) were more likely to select all three non-nutrient criteria, from the provided list of healthfulness indicators.
		*-Ingredients*		
H1b: more likely to prioritize *“Unprocessed or minimally processed”*		-*Processing*	OR: 2.82 (2.03–4.48)*** AOR: 3.01 (2.02–4.48)***	
H1c: more likely to prioritize *“Farming/production practices (pesticides, gmos)*”		*-Farming*	OR: 1.82 (1.30–2.58)** AOR: 1.76 (1.20–2.59)**	
H2: adults reporting efforts to reduce UPF are less likely to prioritize nutrient criteria when evaluating food healthfulness.				
H2a: less likely to prioritize *“Quantity of added sugars”*	*“Made changes in the foods I buy”* to reduce ultra-processed food, compared to *“never heard of”* ultra processed food	Frequency of prioritizing:	OR: 0.59 (0.41–0.83)** AOR: 0.66 (0.45–0.97)*	*H2* mostly supported: adults reporting both concern about food processing and efforts to reduce UPF purchase were less likely to select all of the nutrient criteria, from the provided list of healthfulness indicators. *Food groups* were more equally prioritized than other nutrient-oriented criteria and were not associated with UPF perceptions in bivariate analysis.
		-*Added sugar*		
H2b: less likely to prioritize *“Quantity of sodium/salt”*		-*Sodium*	OR: 0.48 (0.33–0.68)*** AOR: 0.50 (0.33–0.75)**	
H2c: less likely to prioritize *“Quantity of fat, and/or types of fat”*		-*Fat*	OR: 0.51 (0.35–0.73)*** AOR: 0.49 (0.32–0.74)**	
H2d: less likely to prioritize *“Food groups: grains, vegetables, fruits, meat, dairy, nuts”*		-*Food groups*	OR: 0.73 (0.51–1.06) AOR: 0.65 (0.43–0.97)*	

a Adjusted ORs are adjusted for all study variables, including age, annual income, educational attainment, gender, race/ethnicity, and having children in the home. *p < 0.05, **p < 0.01, ***p < 0.001.

When describing the relationships between food and health, the majority of survey respondents (79%) selected at least one non-nutrient criterion, including assessments of the ingredients, and extent of industrial production and processing. This finding is higher than Lusk reported (47.9%) when asking survey respondents in 2019 whether the concept of food healthiness should be “based on more than nutrient content” (25). Our finding that familiar and unprocessed ingredients were often prioritized as indicators of food healthfulness (selected by 44 and 42% of respondents, respectively) is aligned with Belarmino et al. (27), who reported a similarly large number of people (approximately half) citing food processing and whole foods when describing the criteria for use in labeling foods healthy in a national dataset of public opinions on “healthy” food.

We found that 34% of respondents stated that they made changes to avoid UPF, however we also found that 79% of respondents prioritized non-nutrient criteria including assessments of the ingredients and processing. This finding suggests that the majority of people are likely familiar with the concept of ultra processed food, even if they are not familiar with the term. With the increasing popularity of “clean eating” (41, 42) it is quite possible that consumers could already be scanning labels similar to Monteiro’s (9) descriptions of ultra processed food when interpreting food and nutrition labels in the marketplace.

Analysis of demographic variables revealed some statistically relevant groupings. Female respondents more often reported previously hearing of the term ultra processed food and were more likely to prioritize the non-nutrient healthfulness perception indicators. Older respondents more often prioritized nutrient indicators including sodium, as 55.3% of adults in the oldest age category (75+) selected sodium, compared to 41.9% overall. Previous research suggests that women are more likely to read nutrition labels when shopping (37) and age is also related to use of nutrition labels (35, 43) however this prior research rarely compared use of nutrition labels with other types of food labels, so a direct comparison with the present study cannot be made. Existing “clean eating” research has focused on females and younger adult populations (41) and we found that these same groups tended to be more likely to prioritize non-nutrient, food quality attributes, including familiar ingredients, minimal processing, and transparency statements (e.g., non-gmo) when describing the relationships between food and health. Globally, consumers more often pay attention to information about ingredients and processing than nutrients (30) and our findings provide additional evidence that when U.S. consumers are identifying healthy food choices in the marketplace, they are able to prioritize a combination of nutrient and non-nutrient attributes. Our finding that female and younger adults more often prioritized food quality attributes (ingredients, processing, and production practices) while males and older adults more often prioritized nutrient attributes (sodium and fat) demonstrates the range of frameworks for making healthy food choices in different market segments. Combination labels, incorporating nutritional information as well as additional information regarding food production and environmental sustainability indicators could harness a greater number of purchase motivations (44) and our findings could help provide insights into the development of updated food labels incorporating both nutrient and non-nutrient criteria into innovative labeling schemes that are aligned with existing consumer preferences.

There have been calls for global action to target and reduce UPF consumption through policy approaches such as clearly worded dietary guidelines that “unambiguously” describe the need for the public to avoid UPFs (5). It is estimated that 70% of the food products in U.S. supermarkets are ultra-processed (21, 45) and some public health advocates have called for “policies that encourage the production of better and less processed foods, increase the availability, accessibility, and affordability of nutritious minimally processed foods, and restrict the ability to market ultra-processed foods” (3). Policy led reformulations can lead to improvements in food environments—an important determinant of food consumption patterns (46, 47) and help support public health objectives. As an example, the FDA’s mandate to report trans fatty acids on U.S. nutrition labels in 2006 effectively encouraged large-scale industry reformulations which contributed to reductions in dietary trans-fatty-acid levels. It is estimated that this policy change likely prevented 50,000 premature deaths per year (48). Food labels identifying UPF could support similar industry reformulations and support reductions in intake patterns across all population groups, similar to the impacts of trans fat declarations in reducing chronic disease and mortality rates. In addition to these hypothetical reformulation effects, our findings demonstrate consumer interest in additional information on food and nutrition labels regarding the extent of industrial processing, to support food purchasing decisions. Food labels identifying ultra-processed foods have been proposed in prior literature as a public health strategy (3, 5), and our findings suggest that UPF labels could be well aligned with consumer frameworks for identifying healthy foods in the marketplace.

One barrier to action on UPF labels and other policy actions has been a lack of consensus in the nutrition community regarding the health implications of UPF. While there has been a significant body of nutrition research using Nova to identify ultra-processed foods, some have challenged the concept, arguing that it is too broad to be meaningful (49) and that Nova does not contribute meaningfully to existing nutrient-based indexes for assessing diet quality and predicting disease risks (50, 51). A recent analysis demonstrated that it is theoretically possible to follow a healthy diet (defined based on the characteristics of healthy dietary patterns, as described in the 2020 Dietary Guidelines for Americans) when consuming mostly ultra-processed foods (80% of calories or more from UPF) however the authors acknowledged that this study was limited by its theoretical design (52). As future dietary guidelines consider whether to incorporate this UPF concept along with current nutrient recommendations—our findings demonstrate how a “whole food” approach to healthy eating, such as emphasizing Nova group 1 foods, could be more aligned with consumer frameworks for identifying healthier foods, than the nutrient-centric frameworks which have guided previous versions of the Dietary Guidelines for Americans.

When making decisions about food and health, more respondents prioritized food production and farming practices (including use of pesticides and genetically modified organisms), than fat or sodium. While this finding could be an indication of Vermont’s unique agricultural identity (discussed below), evidence of broader support for these same issues was also observed in a global survey conducted by Nielsen Company (30) in 2015 and by Belarmino et al. (27) in a national dataset of public opinions on healthy food labels. These similar findings help confirm the validity of our findings.

Limitations include the use of data from one state (Vermont), which limits the generalizability of our findings. When compared to state estimates of Vermonters, study respondents appeared to be older and report higher educational attainments, trends commonly reported in both telephone and online surveys (53). Vermont leads the nation in the number of farm stands, direct-to consumer sales, and farmers markets per capita (54) resulting in a state with a unique, rich agricultural heritage, potentially resulting in higher levels of knowledge about food production and processing. Vermont was the first and only state to label genetically modified foods (39) and Vermonters may have different opinions on the UPF issue compared to other populations, limiting external validity. While these characteristics limit generalizability, our findings are aligned with recent literature (28, 29) discussing how the concept of UPF as defined by Nova is related to the criteria and frameworks that people use when describing food-health relationships. Data were collected in 2022, and the UPF issue has been more discussed in the media since the 2024 U.S. Federal elections, making it likely that even people have heard of this term in 2025 when compared to 2022. Future research is needed to validate these findings in other, more diverse populations.

It is possible that additional criteria may be more important to some consumers than the criteria assessed in this survey. We did not perform validity testing for the key outcome variable (perceived healthfulness priorities), instead we assumed that respondents would be able to comprehend our list of seven health-oriented food attributes, and that our list (which was developed based on consumer nutrition trends, including so-called “clean eating”) would contain the most important attributes for assessing healthfulness. Additional threats to internal validity include not applying correction for multiple testing, the small subgroup sizes which could potentially undermine the stability of regression estimates, and our decision to combine together respondents who were “unsure/do not know” whether they previously heard of UPF with those who have not previously heard of the term, a potential source of measurement error in this grouping variable. Sensitivity analysis (Supplementary Table S1) confirmed the decision to examine ultra processed food perceptions as four response categories, as the second model with five response categories did not improve from the main model (four response categories), a finding which helps justify our decision to combine those who were both “unsure” and “never heard of” UPF.

According to a 2010 Institute of Medicine report on front of package nutrition labeling, nutrition labels need to be “flexible enough to accommodate continuing advances in science and nutrition as well as changes in consumer behavior” (55). In 2025, the FDA for the first time reported working towards a definition of UPF, and the FDA and USDA are also seeking public comments to help develop a uniform definition of UPF “to pave the way for addressing (the) health concerns associated with the consumption of UPFs” (21). When making decisions about food and health, our findings suggest that many adults living in Vermont are likely assessing a much broader range of criteria than what is reported on existing nutrition labels, including assessments of ingredients, production and processing- which are characteristics of UPF. Our findings suggest that many food consumers are likely to be concerned about the health implications of UPF and would be receptive to advice on this topic in future versions of the Dietary Guidelines for Americans.

## 5 Conclusion

This study demonstrates the existence of consumer strategies for identifying and avoiding ultra processed foods in the marketplace. The public considers a range of nutrient and non-nutrient criteria when describing the relationships between food and health, including criteria related to industrial processing. In the present study, we found that one third (34%) of respondents explicitly reported efforts to reduce UPF consumption, however, we also noted larger proportions (79%) prioritizing UPF attributes including assessments of the list of ingredients, and production and processing attributes, when describing the characteristics of healthy foods, suggesting that an even greater proportion of the public is likely concerned with the health implications of UPFs, even if they have not heard of the specific term.

Many people in the U.S. are concerned with the health implications of ultra processed food and are looking beyond nutrition labels to assess the extent of industrial production and processing when making decisions about food and health. Food labels (or other educational efforts) have been proposed as a strategy to support consumers in identifying ultra processed foods, and our findings demonstrate how future UPF labels could be well aligned with existing consumer frameworks and strategies for identifying healthy foods in the marketplace. Labels identifying ultra processed foods could also support larger public health priorities such as encouraging industry reformulations that make it easier for everyone to identify and prioritize more wholesome, minimally processed foods.

## Data Availability

The raw data supporting the conclusions of this article will be made available by the authors, without undue reservation.

## Supplementary material

SUPPLEMENTARY FIGURE S1 Conceptual overview of the nutrient and non-nutrient healthfulness perception indicators included in the study and the general study hypothesis that people who avoid ultra-processed foods are more likely to prioritize non-nutrient attributes when identifying healthy foods in the marketplace.
