# Supplementary figures and images for: Perceptions of ultra processed food are associated with strategies for identifying healthy foods in online survey of adults living in Vermont
Source: Front Public Health. 2025 Nov 4;13:1679616. doi: 10.3389/fpubh.2025.1679616 (PMC12623193; doi:10.3389/fpubh.2025.1679616)

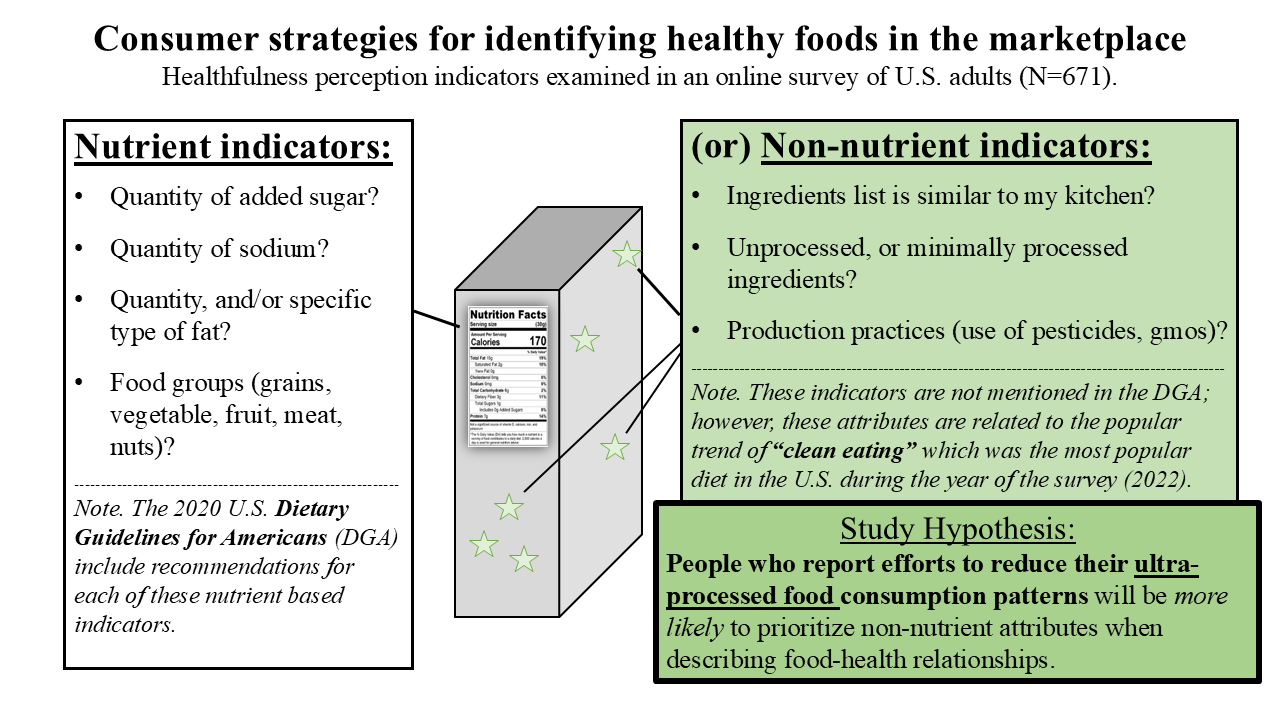

Supplement: SUPPLEMENTARY FIGURE S1 — Conceptual overview of the nutrient and non-nutrient healthfulness perception indicators included in the study and the general study hypothesis that people who avoid ultra-processed foods are more likely to prioritize non-nutrient attributes when identifying healthy foods in the marketplace. [file Image_1.tif]
