# Supplementary material for: Perceptions of ultra processed food are associated with strategies for identifying healthy foods in online survey of adults living in Vermont
Source: Front Public Health. 2025 Nov 4;13:1679616. doi: 10.3389/fpubh.2025.1679616 (PMC12623193; doi:10.3389/fpubh.2025.1679616)
Supplement: Supplementary file 2 [file Table_1.docx]

Supplemental Table. Results of sensitivity analysis comparing associations between variables in a second model, which classified ultra processed food perceptions as five categories (instead of four) by separately analyzing those who reported “don't know” and “never heard of” ultra processed food into separate response categories (N=671).

|  | **Model 2^+^** | | | | | | ***Main Model*** |
| --- | --- | --- | --- | --- | --- | --- | --- |
| *Criteria selected for describing healthy foods* | Unsure  7.8% (52) | Never heard of UPF  40.2% (270) | Not concerned about UPF  4.8% (32) | Concerned about UPF  13.7% (92) | Made changes to reduce UPF  33.5% (225) | Results of  chi-square tests: | |
|  | % (n) | % (n) | % (n) | % (n) | % (n) | **Model 2**  χ^2^ (4) | *Model 1*  *χ^2^ (3)* |
| Nutrient criteria | | | | | | | |
| -Quantity of added sugar | 55.8 (29) | 57.4 (155) | 50.0 (16) | 58.7 (54) | 44.0 (99) | 10.845* | *10.798** |
| -Quantity of sodium | 51.9 (27) | 47.4 (128) | 40.6 (13) | 43.5 (40) | 30.7 (69) | 17.257* | *16.890** |
| -Quantity, and or specific type of fat | 46.2 (24) | 42.2 (114) | 18.8 (6) | 47.8 (44) | 27.6 (62) | 22.752* | *22.464** |
| -Food groups (grains, vegetable, fruit, meat, nuts) | 25.0 (13) | 40.0 (108) | 50.0 (16) | 38.0 (35) | 30.7 (69) | 10.281* | *6.019* |
| Non-nutrient criteria | | | | | | | |
| -Ingredients list is similar to my kitchen | 38.5 (20) | 40.0 (108) | 50.0 (16) | 33.7 (31) | 54.7 (123) | 17.143* | *17.101** |
| -Unprocessed, or minimally processed ingredients | 30.1 (16) | 33.3 (90) | 46.9 (15) | 37.0 (34) | 58.7 (132) | 37.602* | *37.484** |
| -Production practices (use of pesticides, gmos) | 44.2 (23) | 35.9 (97) | 40.6 (13) | 32.6 (30) | 52.0 (117) | 16.798* | *15.561** |

Note. The full descriptions of each UPF response category are: *“i) I have never heard of the term ultra-processed food; ii)I have heard of ultra-processed food, and I am not concerned with the level of food processing of the foods that I but for my household; iii) I have heard of ultra-processed food and I am concerned about the level of food processing in the foods I eat, but I have not made changes in the foods I buy for my household; iv) I have heard of ultra- processed food and I am concerned about the level of food processing in the foods I eat and I have made changes in the foods I buy for my household; v) don’t know/unsure.”*

+Model 2 classified ultra processed food perceptions as five categories (instead of four) by separately analyzing those who reported “don't know” and “never heard of” ultra processed food into separate response categories.

*Indicates significant associations (p<.05), reported p-values represent differences in observed frequencies (across rows) between response categories (either 4 or 5 categories), based on Pearson chi-square test (two sided).
